# Cognitive warfare: a conceptual analysis of the NATO ACT cognitive warfare exploratory concept

**DOI:** 10.3389/fdata.2024.1452129

**Published:** 2024-11-01

**Authors:** Christoph Deppe, Gary S. Schaal

**Affiliations:** Helmut-Schmidt-University/University of the Federal Armed Forces, Hamburg, Germany

**Keywords:** cognitive warfare, disinformation, NATO, hybrid threats, concept analysis

## Abstract

This study evaluates NATO ACT's cognitive warfare concept from a political science perspective, exploring its utility beyond military applications. Despite its growing presence in scholarly discourse, the concept's interdisciplinary nature has hindered a unified definition. By analyzing NATO's framework, developed with input from diverse disciplines and both military and civilian researchers, this paper seeks to assess its applicability to political science. It aims to bridge military and civilian research divides and refine NATO's cognitive warfare approach, offering significant implications for enhancing political science research and fostering integrated scholarly collaboration.

## 1 Introduction

Cognitive warfare is an emerging academic and military concept that aims to address the exploitation of human cognition and technology to disrupt, undermine, influence, or modify human decision-making [see Claverie and du Cluzel, 2021; du Cluzel, 2021; Reding and Wells, 2022; Deppe, 2023]. It has become increasingly relevant in the current security environment, where adversaries continuously seek to undermine the integrity of political processes in democratic societies, as well as their military-strategic objectives, by deploying sophisticated strategies through coordinated political, military, economic, and information efforts [see Backes and Swab, 2019; Splidsboel Hansen, 2021; Hung and Hung, 2022; Bernal et al., 2020; Adlakha-Hutcheon et al., 2023; Miller, 2023]. Cognitive warfare has roots in early strategies of manipulation and deception in political and military contexts, exemplified by tactics from ancient times, such as those of Sun Tzu. Strategies evolved as information dissemination technologies advanced, broadening its focus from decision-makers to entire populations by leveraging psychological operations, propaganda, and most recently the cyber domain. Today, it encompasses the strategic use of neuroscience, behavioral science, and digital technologies to influence and disrupt human cognition, making it a pivotal element of modern conflict and strategic competition.

Several definitions of cognitive warfare have been published in the literature. Hung and Hung (2022) offer a minimalist conceptualization of cognitive warfare, arguing that it is not a standalone concept but rather a subordinate concept of hybrid warfare and entangled with other traditional non-kinetic warfare forms, such as information warfare and cyber warfare. Cognitive warfare is hereby distinguished by its distinct focus on cognitive effects. Hung and Hung also conceptualize Chinese cognitive warfare as operations that aim to control others' mental states and behaviors by manipulating environmental stimuli. The Chinese approach to hybrid warfare is also based on the Three Warfares concept, which is a combination of psychological warfare, public opinion warfare, and legal warfare (Lee, 2014).

Bernal et al. (2020) differentiate cognitive warfare from information warfare by delineating their distinct objectives: whereas information warfare centers on controlling the dissemination of information, cognitive warfare strategically aims to shape and manage the reactions of individuals and groups to that information. Backes and Swab (2019) offer a similar view with a general definition: “Cognitive warfare is a strategy that focuses on altering how a target population thinks—and through that how it acts”. In relation to Russian information warfare tactics, Tashev et al. (2019) highlight the relevance of the cognitive domain, which they consider to be the relevant aspect of information warfare. Splidsboel Hansen (2021) also illustrates the relevance of a variety of information operations in Russian efforts to influence the cognitive domain. More generally in the Russian approach to warfare, information operations and other measures aimed at reaching cognitive effects are a small number of possible measure among a larger portfolio which includes “all means of national power” (Bartles, 2016). Miller (2023) seeks to distinguish cognitive warfare from related concepts such as cyberwar, cyber conflict and others by referencing four dimension of harm: physical or psychological harm to humans, damage to physical objects, damage to software and data and lastly harm to institutions. Miller sees cognitive warfare as a form of conflict that inflicts psychological harm on individuals and damages institutions. He positions it as a conflict that operates below the thresholds of conventional war and covert operations, thus introducing the nuanced concept of covert cognitive warfare, which subtly undermines its targets without escalating to open hostilities. This view clashes with other conceptualizations of cognitive warfare, that include activities below and above the threshold of war and also kinetic activities as a means to reach cognitive effects (NATO Allied Command Transformation, 2023). A technical evaluation report on a NATO Science and Technology Organization (STO) workshop, which was aimed to contribute to a common understanding of cognitive warfare narrowed the interdisciplinary perspectives from military and academic backgrounds down to include the following components: “the use of technology enabled tactics, techniques, procedures and tools to influence human decision-making at an individual and/or societal level (…) altering human behavior to align with an adversaries' political, social, economic, or military objectives (…) means (i.e., training, technology, policy) to defend and secure the cognitive battlespace (…) resilience and a whole-of-society perspective” (Adlakha-Hutcheon et al., 2023).

NATO Allied Command Transformation (ACT) is tasked with developing a comprehensive military cognitive warfare concept, which will ultimately be part of NATO doctrine. A final version of the cognitive warfare concept is projected to be finalized in late 2024 (NATO Allied Command Transformation, 2023). The cognitive warfare concept by NATO ACT is the most comprehensive attempt to produce a concept under this term to date. Some existing cognitive warfare conceptualizations are subject to conceptual shortcomings, such as unclear definitions, overspecification, conceptual stretching, concept travel etc., which are also due to different conceptual objectives. A unified understanding of a scientific cognitive warfare concept has not yet been reached. This is attributable in part to the nascent nature of the field, but also to the complexity of this interdisciplinary subject area, compounded by numerous technological innovations critical to cognitive warfare. Furthermore, the recognition of a sixth domain of warfare is a different discourse that continues to evolve (Allen and Gilbert, 2010). Because of a number of evolving challenges in global security and information environments there is an ongoing discussion whether a cognitive or human domain could be a sixth domain of warfare (Le Guyader, 2022). However, while notable differences between existing conceptualizations exist, for the purpose of this work, also drawing from proposed NATO definitions, the following working definition of cognitive warfare is used: Cognitive warfare is a tactic, which combines traditional and emerging technologies as well as measures above and below the threshold of war to achieve cognitive effects in an adversary's population, as well as in their political and military leaders. The definition builds upon three key attributes, that need to be present to classify a given attack as cognitive warfare. First, the attack needs to seek cognitive effects, meaning an attempt to alter the cognition of the targets must be present. Second, an element of warfare, meaning a hostile power competition with covert or overt measures above or below the threshold of war. And third, the use of technological means to amplify and/or enable cognitive attacks and their effects. It might be especially relevant for research applications in the sciences, that the attribute technology may also serve to functionally distinguish cognitive warfare from neighboring concepts like hybrid warfare.

It is important to note that the cognitive warfare concept within NATO has been developed as a military concept, to fulfill specific functions within NATO doctrine. Also, the practice and aim of military concept development in NATO differs greatly from concept development in the social sciences (NATO Allied Command Transformation, 2021). While military concepts usually describe new capabilities in the military context, social science concepts aim to produce analytically valuable building blocks, which can be connected to existing theories and be integrated in feasible research designs. The term “cognitive warfare” appears extensively in scientific literature, occasionally in the context of analyzing contemporary conflicts. However, definitions and conceptualizations of cognitive warfare vary significantly across these publications. A critical observation of the existing literature reveals a distinction between conceptualizations of cognitive warfare in scientific literature and contributions in the context of NATO. In the first body of literature, cognitive warfare is frequently viewed as a narrower concept, often subsumed under broader categories such as hybrid warfare. In the latter, NATO's conceptualizations tend to portray cognitive warfare as a comprehensive, standalone concept with broader strategic implications. Therefore, examining NATO ACT's exploratory concept of cognitive warfare will notably improve mutual intelligibility between the different strands of research.

NATO ACT's cognitive warfare exploratory concept represents the most thorough effort to date in formulating a cognitive warfare framework, incorporating contributions from a wide array of both military and civilian researchers within the context of the NATO Science & Technology Organization (STO). Given the extensive collaborative effort behind this concept, it warrants a thorough examination to assess its relevance to the social sciences and to determine whether, despite potential immediate limitations, it offers any value for future research and practical applications. This study also aims to reduce the conceptual unclarity between military and academic conceptualizations of cognitive warfare and to enhance the development of the concept within both fields of application. Additionally, it seeks to delineate the relationship of cognitive warfare more precisely to adjacent concepts such as FIMI and hybrid threats or hybrid warfare. The lack of clearly defined intensions and extensions of the concept may hinder the understanding of their functional differences, analytic capacity, and interoperability (Sartori, 1970). Therefore, it is of utmost importance to be aware of conceptual mismatches between academic and NATO (military) concepts. The later could hamper the above-mentioned superiority resulting from the connection between academia and NATO. Furthermore, concepts such as cognitive warfare are significant not only in scientific and military contexts but also in political communication. It is probable that cognitive warfare will be employed to engage with the political and public spheres, explaining and justifying research, military actions, and ultimately policy decisions. This pattern has previously been observed with the concept of hybrid threats.

This leads to the following research questions: What are the analytical strengths and weaknesses of the cognitive warfare exploratory concept by NATO ACT from a political science standpoint, and how can cognitive warfare be integrated into existing academic and political conceptual landscapes?

## 2 Cognitive warfare—Background and organizational considerations

Over the past two decades, the delineations between peace and conflict have become increasingly indistinct. In the same period, a rapid change fueled by technological innovation has radically transformed the way individuals, groups, institutions, and whole societies communicate, how they produce and consume information. The effects of hybrid tactics, such as disinformation, can be observed in many democratic societies. Many activities are attributed to Russia and China (Splidsboel Hansen, 2021; Hung and Hung, 2022; Hellström et al., 2024). A prominent case of state intervention in democratic proceedings was the Russian involvement in the 2016 U.S. presidential election, favoring Republican candidate Donald J. Trump. The specifics of Russian action during this election were outlined in a report compiled by Special Counsel Robert Mueller (2019). Within the EU the European Union External Action Service (EEAS) has registered a significant prevalence of Foreign Information Manipulation and Interference (FIMI) and disinformation, particularly highlighted by Russia's actions during its invasion of Ukraine. The Kremlin strategically used disinformation to justify its actions and manipulate international opinion, leading to unprecedented EU responses, including sanctions against Russian outlets like RT and Sputnik (European Union External Action, 2023b; Deppe and Schaal, 2022). For instance, the EEAS detected almost 400 FIMI incidents in 2022, demonstrating the extensive and coordinated efforts of foreign actors to undermine EU stability (European Union External Action, 2023b). Another example is the EUvsDisinfo initiative by the EEAS, which has documented over 13,300 cases of pro-Kremlin disinformation as of December 2021, revealing a systematic effort to manipulate public opinion within the EU (European Union External Action, 2021). Additionally, China has been implicated in disinformation campaigns that target the EU, particularly concerning COVID-19, where state-controlled media spread false information to counter criticisms of China's handling of the pandemic (European Union External Action, 2023a).

The emergence of artificial intelligence (AI) heralds significant changes in how information is managed and disseminated. On the positive side, AI systems, readily accessible and operable without specialized knowledge, offer unparalleled access to resources, information, and knowledge, potentially enriching decision-making processes by streamlining complex data analysis. Additionally, the capacity for personalization that AI brings can enhance user engagement with information platforms.

Conversely, the advent of AI introduces several challenges. AI models present a high potential for misuse, notably in the mass production of spurious media and disinformation. This capability could profoundly destabilize information ecosystems. Moreover, the risk of exacerbating digital divides looms large, as entities equipped with advanced AI tools could gain disproportionate influence over public discourse and perceptions. Furthermore, while personalization can improve engagement, it also raises significant concerns regarding privacy breaches, targeted influencing of groups and individuals as well as the creation of echo chambers, thereby limiting exposure to diverse viewpoints.

This transformation, along with changes in economic systems, the global security environment and other factors has made it necessary to develop new academic and military concepts to make sense of and analyze novel forms of competition and conflict situations. These concepts include unconventional warfare, hybrid threats, hybrid warfare, and FIMI, to name a few. More recently, considering drastic changes in the global security environment and the rapid emergence of new threats in the cyber and cognitive domains, the concept of cognitive warfare has been introduced to academic and military discourses. Like with any other newly formulated concept, it has to be made sure, that the emerging cognitive warfare concept adds actual analytic utility to diverse research applications, meaning the concept can actually be used to measure a new phenomenon that is not or incompletely captured by existing concepts.

### 2.1 The purpose of the cognitive warfare concept within NATO

In the 2022 strategic concept (NATO, 2022) NATO highlights activities by Russia and China as significant threats to the Alliance's security and interests. It points out that Russia employs tactics such as coercion, subversion, aggression, and annexation to establish spheres of influence and direct control. The country utilizes a combination of conventional, cyber, and hybrid means against NATO and its partners. Regarding China, the strategic concept notes that its stated ambitions and coercive policies challenge NATO's interests, security, and values. China employs various political, economic, and military tools to expand its global presence and assert influence (Hung and Hung, 2022; Aukia and Kubica, 2023). However, it maintains opacity about its strategy, intentions, and military build-up. The strategic concept 2022 states that China's aggressive use of hybrid and cyber operations, along with its confrontational rhetoric and dissemination of disinformation, target Allies and pose a threat to Alliance security.

The strategic concept also recognizes the escalating threat of hybrid tactics (NATO, 2022). These tactics encompass a spectrum of measures, including political, economic, energy, and informational methods, employed coercive to attain strategic goals (Aho et al., 2023). Notably, in the strategic concept 2022 NATO acknowledges that, hybrid operations against Allies can escalate to the level of an armed attack, potentially necessitating the invocation of Article 5 of the North Atlantic Treaty (NATO, 2022, 1949). Consequently, NATO aims to improve its capabilities for preparedness, deterrence, and defense against the coercive application of hybrid tactics, by state or non-state actors. The strategic concept also states that the alliance will maintain its support for partners in countering hybrid challenges, striving for optimal collaboration with relevant institutions like the European Union. From this brief analysis of the strategic concept, one of the most important policy documents within NATO, it can be concluded, that hybrid tactics and related activities are a severe vector to NATO, which demands adequate responses. The NATO Warfighting Capstone Concept (NWCC) is subordinate to the strategic concept and is a military concept that outlines NATO's vision for maintaining and developing its military advantage through 2040 (NATO, 2021). It addresses the changing character of war and power competition and emphasizes the need for NATO to be able to operate in all domains, including space and cyberspace. The NWCC also highlights the importance of interoperability and partnerships with other nations and organizations. The NWCC defines “6 Outs”, which are a set of functions that the future Alliance MIoP (Military Instrument of Power) must aspire to outperform to maintain NATO's military advantage. These functions are: out-think, out-excel, out-fight, out-pace, out-partner, and out-last (NATO, 2021). The NWCC posits that by achieving superiority in these domains, NATO will enhance its capability to comprehend and counter potential activities by adversaries, cultivate partnerships, and adjust to evolving situations. The resulting warfare development imperatives are five key areas on which NATO must concentrate to achieve these goals. These imperatives are: cognitive superiority, layered resilience, influence and power projection, cross-domain command, and integrated multi-domain defense (NATO, 2021).

Within the five warfare development imperatives listed in the NWCC, the cognitive warfare concept is most relevant in cognitive superiority. Cognitive superiority describes the ability of NATO to better understand the operating environment and potential adversaries relative to its own capabilities and objectives (NATO, 2021). It involves expanding knowledge and understanding across all domains, enabled by technology, to maximize the ability of military leaders to anticipate, think, decide, and act. The goal is to achieve a cognitive advantage over potential adversaries by building better situational awareness and understanding. Hereby, the cognitive warfare concept within NATO doctrine will serve a 2-fold purpose: to improve the comprehension of evolving threats within the cognitive realm and to lay the groundwork for potential future developments in warfare in the cognitive domain (NATO Allied Command Transformation, 2023). The concept shall provide a unified framework for comprehending and effectively addressing cognitive warfare, outlining its dynamics, mechanisms, and implications for both NATO's warfighting capabilities and cognitive superiority. Its overarching objective is to improve NATO's cognitive resilience, as well as to protect and enhance decision-making capacities.

In essence, the cognitive warfare concept shall enhance NATO's understanding of upcoming cognitive threats, protect cognitive resilience by defining potential impacts, and produce a holistic strategy for mitigating the effects of adversarial cognitive warfare through tactics like education, collaboration, protection, and influence in the cognitive domain. This concept thus fulfills a distinct role as a lower-level military concept, positioned subordinate to the NWCC. In due course, the aspiration is for the cognitive warfare concept to be integrated into NATO's official doctrine (NATO Allied Command Transformation, 2023; Groestad, 2022).

## 3 The cognitive warfare exploratory concept

In this chapter, the cognitive warfare exploratory concept, published by NATO Allied Command Transformation (ACT), is reviewed from a methodological perspective from the social sciences. As of spring 2024 it is the latest published non-draft version of the NATO ACT cognitive warfare concept. In this analysis, focus is placed solely on elements of the exploratory concept that are analytically valuable, omitting detailed discussions of technological, legal, or organizational specifics.

### 3.1 Basic concept and definitions

The review of the cognitive warfare concept begins at the highest level, with the concept term and the basic definition. The proposed definition of cognitive warfare as published in the exploratory concept by NATO ACT is “Activities conducted in synchronization with other Instruments of Power, to affect attitudes and behavior by influencing, protecting, or disrupting individual and group cognition to gain advantage over an adversary” (NATO Allied Command Transformation, 2023).

The substantive necessity for a concept like cognitive warfare is derived from observed challenges for NATO, which can be broken down into two developments. First, technological progress and shifts in information consumption, wield adversaries' greater capacity to amass and manipulate data, sway emotions, and shape beliefs and behaviors. This enables the leveraging of societal divisions using novel technologies [e.g., artificial intelligence (AI), emerging & disruptive technologies, data harvesting], and the proliferation of social media influencing individuals' thoughts, emotions, and actions. As the authors themselves state, this mirrors hybrid warfare tactics, where adversaries target society as the vector to exert influence indirectly on key targets: political and military leaders. The effectiveness of influence campaigns is conceptualized to hinge on the calculated manipulation of emotions and cognitive predispositions to instigate widespread shifts in attitudes and behavior. These alterations are frequently nuanced, blurring the distinction between genuine societal debate and discord, and the hostile exploitation of societal divisions through cognitive attacks (NATO Allied Command Transformation, 2023).

Second, it is stressed that cognitive attacks are not new, however the concept defines them as deliberate offensive maneuvers aimed at influencing perceptions, beliefs, interests, decisions, and behavior by directly targeting the human mind. It is conceptualized that the innovation lies in the adversaries' ability to rapidly and anonymously carry out cognitive attacks within the Information Environment (IE) using digital platforms and emerging disruptive technologies (EDTs) (NATO Allied Command Transformation, 2023).

The focus on cognition and the human mind distinguishes the cognitive warfare concept from other concepts like hybrid threats, FIMI and disinformation, whose conceptualized effects often end at the acceptance or rejection of specific information or narratives. In cognitive warfare, the main effect of activities is conceptualized to lie in the manipulation of emotional and subconscious processes of the human mind. This is much more far-reaching than other related concepts like Foreign Information Manipulation and Interference (FIMI) or hybrid threats. The authors clarify that, “synchronized and coordinated attacks on emotions, thoughts and behaviors impact will, morale, decision-making and situational understanding” (NATO Allied Command Transformation, 2023). Furthermore, “Cognitive attacks are designed to use information to activate the subconscious processes in our brains, making it difficult for our conscious minds to perceive the presence of a cognitive threat” (NATO Allied Command Transformation, 2023). These factors constitute an approximation to the empirical referents that partly constitute the extension of the cognitive warfare concept.

### 3.2 Problem space

The problem space section of the exploratory concept provides an overview of the potential dangers posed by cognitive warfare, again adding to the empirical referents of the concept. It begins by labeling cognitive warfare to be a value-neutral set of tactics, which may be employed at every stage on the continuum of competition. It is furthermore problematized as a Whole-of-Society Problem in which “adversaries are targeting the NATO Alliance through campaigns to malignly influence the attitudes, decisions and behaviors of individuals, groups and societies. Emerging and Disruptive Technologies (EDTs) and sciences enable these cognitive attacks. Our adversaries aim to turn our strengths into vulnerabilities that weaken the Alliance” (NATO Allied Command Transformation, 2023). In this definition the role of technological innovations in the distribution of influence campaigns is highlighted as a powerful enabling factor, that can be used to attack the discourse spaces of open liberal democratic societies. Furthermore, the military challenge of cognitive warfare is described as “Alliance decision-making, mission and forces are directly and indirectly vulnerable to cognitive attacks. The role of the Military Instrument is the cognitive dimension is unclear, particularly below the threshold of armed conflict. This causes gaps in policy, defense planning and capabilities” (NATO Allied Command Transformation, 2023). Hereby, the concept is connected to ongoing discussions in many democratic societies, about the role of different governmental institutions in the mitigation of threats in the information environment.

Next, the kind of actions, that are considered to be part of cognitive warfare are listed. These tactics, or vectors and enablers can be the defining attributes that constitute the meaning or intension of the cognitive warfare concept^1^. Cognitive attacks, both presently and potentially in the future, are described to be facilitated through a range of vectors, capabilities, and enablers:

*Traditional vectors and enablers:*

This category includes kinetic force and established channels like broadcast and print mass media. Additionally, it involves various actors such as corporate, state, and political entities, along with interpersonal engagement (NATO Allied Command Transformation, 2023).

*Existing technology vectors and enablers:*

This domain leverages contemporary technology. It encompasses social media platforms, the utilization of big data, the integration of augmented reality and wearable smart devices, as well as the use of gaming and encrypted communication platforms. Avatars and virtual profiles are also instrumental in this context (NATO Allied Command Transformation, 2023).

*Emerging technology vectors and enablers:*

This category delves into cutting-edge technologies that hold significant potential for cognitive attacks. It encompasses synthetic media, exemplified by deepfakes and AI-driven media. Additionally, it includes the widespread use of artificial intelligence, the immersive realm of the Metaverse, and the concerning emergence of neuroweapons. The listed vectors and enablers encompass a wide spectrum of tactics and approaches, underscoring the wide intension of the cognitive warfare concept (NATO Allied Command Transformation, 2023).

Further, the authors identify various individual risk factors and resulting triggers that heighten susceptibility to micro-level cognitive attacks. These include deficiencies in accurate knowledge, deeply ingrained worldviews, negative emotional experiences, and limited literacy (NATO Allied Command Transformation, 2023). Addressing these factors is considered crucial for enhancing the resilience of both NATO personnel and member nations against cognitive attacks. This can be achieved by improving knowledge, critical thinking, and emotional resilience. Additionally, the document outlines three primary triggers influencing vulnerability to influence and manipulation. These are cognitive inflexibility, the need for social belonging, and emotional arousal. Mitigating these triggers is vital for bolstering resilience against cognitive attacks. This involves promoting cognitive flexibility, fostering a sense of belonging, and managing emotional arousal.

The authors also name risk factors that heighten vulnerability to cognitive attacks on the meso-level i.e., in social and cultural groups. These factors include group polarization and social trust (NATO Allied Command Transformation, 2023). Group polarization, influenced by human social tendencies, can be exacerbated by social media platforms through algorithms that reinforce existing beliefs. This fosters confirmation bias and the spread of disinformation, undermining trust and manipulating groups. Social trust, crucial for societal cohesion, can be exploited by malign actors spreading disinformation to erode trust in institutions and leaders. Addressing these risk factors is conceptualized to be crucial for enhancing the resilience of NATO personnel and member nations against cognitive attacks. This may be achieved by promoting critical thinking, bolstering social trust, and countering group polarization.

Finally, the authors also examine risk factors on the macro level, i.e., societies and nations. The concept highlights nations' varying susceptibility to cognitive attacks, a crucial consideration for NATO in evaluating member nations' resilience (NATO Allied Command Transformation, 2023). While NATO's MIoP does not possess a direct mandate to address these factors, their understanding remains essential. They are conceptualized as the basis for collaborative efforts with NATO partner nations and non-NATO organizations.

In liberal democracies, a notable vulnerability exists to adversarial cognitive warfare, challenging NATO's foundational values (NATO Allied Command Transformation, 2023) as well as democratic core values. While preserving the liberal democratic system remains a priority for NATO, it is recognized as a risk factor due to the principled rejection of authoritarian control methods. Adversaries perceive this as a significant vulnerability that can be exploited to sow discord within societies and erode the ability to govern in line with liberal democratic principles (Deppe, 2023).

Furthermore, the authors name information and media literacy as an important risk factor, as research indicates a direct correlation with susceptibility to disinformation and cognitive manipulation (NATO Allied Command Transformation, 2023). Citizens, including NATO personnel, often remain unaware of their own vulnerabilities to cognitive manipulation. Therefore, the authors underscore the necessity for heightened information and media literacy efforts to counter cognitive manipulation attempts.

Civic engagement encompasses activities that enhance community wellbeing through political and non-political means, breaking down barriers and augmenting societal resilience (NATO Allied Command Transformation, 2023). While improving civic engagement falls beyond NATO's political and military scope, recognizing its protective potential offers an opportunity for the Alliance to collaborate with external entities focused on fortifying societal resilience.

The authors state that, NATO has observed a surge in anti-establishment populism, indicating discontent with prevailing economic, social, and cultural conditions in numerous NATO member nations (NATO Allied Command Transformation, 2023). In some instances, the growing support for populist ideologies may also signify the influence and success of cognitive attacks by adversaries. These are achieved through various means, including espionage, hacking, disinformation campaigns, and covert funding of political movements.

From a methodological viewpoint, the risk factors for an increased vulnerability to cognitive attacks on the micro, meso and macro levels, listed above, can potentially be attributed to be part of the extension of the concept. This is because these factors can be read as a list of variables, that constitute a case, that would be captured by the concept cognitive warfare. From the counter perspective, if a given hypothetical case featured none of the listed risk factors, it would not be captured by a measurement that captures instances of cognitive warfare.

In a subsequent section, the exploratory concept provides a list and description of the intended effects of cognitive warfare (NATO Allied Command Transformation, 2023). Cognitive warfare is conceptualized to entail a diverse range of intended effects, posing intricate challenges in recognizing attacks and their protracted consequences. In this context, cognitive attacks are employed within broader geopolitical strategies to hinder decision-making processes, erode national or institutional unity, sow societal division, exploit identities and narratives, and undermine the resolve to engage in conflict.

First, under “Impede Decision-Making and Disrupt OODA Loop,” (NATO Allied Command Transformation, 2023) decision-making, contingent on information availability, becomes susceptible to manipulation by state and non-state actors. Disinformation compounds uncertainty or propagates false narratives, thereby influencing decision-makers across strata. As an illustration of efforts to undermine decision-making through disinformation, the authors cite Russia's Reflexive Control (RC) theory. The RC theory is conceptualized with the objective of impeding NATO's decision-making processes.

Second, “Divide and Polarize Society” (NATO Allied Command Transformation, 2023) pertains to deep-seated societal polarization, imperiling democracy. Adversaries exploit disinformation to systematically erode social trust, weaken institutions, and impede efforts to reconcile conflicting values and interests. This leads to societal segmentation based on various criteria.

Third, “Weaponize Identity” (NATO Allied Command Transformation, 2023) emphasizes the pivotal role of identity in cognitive warfare, influencing connections to others, societal roles, and cultural and national affiliations. Understanding the potential weaponization of identity is crucial, especially when safeguarding NATO personnel against targeted cognitive attacks.

Next, “Weaponize Narratives” (NATO Allied Command Transformation, 2023) highlights how historical memory and heritage significantly shape individual and group identities, influencing the narratives employed to depict how individuals, communities, and nations perceive themselves. Adversaries adeptly manipulate, discredit, and alter narratives to align with their strategic objectives.

Last, “Impact the Will to Fight” (NATO Allied Command Transformation, 2023) underscores that effective cognitive warfare requires seamless synchronization and coordination to manipulate human cognition, influencing decision-makers' comprehension of the information environment and their resolve to engage in conflict. Cognitive attacks introduce friction within military leadership, potentially eroding trust in NATO leadership and the overarching alliance mission among military personnel over time.

The intended effects of cognitive warfare listed above can best be attributed to the intension of the concept, because they illustrate what an adversary seeks to accomplish by employing measures and tactics conceptualized as part of cognitive warfare. Analytically, “intension” is very difficult to operationalize, however, it is frequently used in related concepts like disinformation (see Wardle and Derakhshan, 2017) or FIMI (see European Union External Action, 2023a). This concludes the review of areas within the exploratory concept that are relevant to the concept specification of cognitive warfare from a methodological perspective.

## 4 Concept analysis

### 4.1 Methodology and data

The definition, use, and significance of concepts in social science is a complex and contested research area (Sartori, 1970, 1984; Collier and Mahon, 1993; Gerring, 2001). In comparative research within the social sciences and beyond, clear and comprehensible concepts are paramount to ensure communicability and intelligibility (Sartori, 1984). In its core form, a referential concept consists of a term, that names the concept; one or more empirical referents, that are captured by the concepts, thereby defining the denotation or extension of a given concept; and lastly, one or more defining attributes, that fill the concept with meaning, defining the connotation or intension of a given concept (Sartori, 1984; Gerring, 2001). Concepts serve as the foundation of the scientific process, informing research questions and hypotheses; they are essential to the development of research designs and to many downstream tasks of research, such as operationalization and research communication. The definition of concepts represents a fundamental preliminary step in the planning of a research endeavor. “When a concept is formulated (or reformulated) it means that one or all of the features is adjusted. Note that they are so interwoven that it would be difficult to change one feature without changing another. The process of concept formation is therefore one of mutual adjustment” (Gerring, 2012). In order to improve the integration and analytic capacity of concepts in applied research, Gary Goertz has introduced to basic concept model. This is a complex structure that enables the use of complex concepts, considering their multidimensional and multilevel properties (Goertz, 2002). The basic concept consists of three levels. First, the basic level, which is the concept identifier as used in hypotheses and theories. Second is the secondary level which includes the concepts defining features or attributes. Third is the data indicator level, which describes what specific data is indicative of the presence of a given attribute. Items on level three are therefore indicative of items on level two, whereas level one and level two share an ontological relationship (Goertz, 2002). The items on the levels two and three can be aggregated in different modes to suit concept meaning and measurement considerations. Goertz (2002) notes that the model can be read top-down when referring to the conceptualization and semantics of a given model, as well as bottom-up when considering the measurement and numerics. The strengths of the model lie in making complex theoretical concepts measurable. The basic concept model is therefore used below produce a concept of cognitive warfare, based on the concept by NATO ACT which can be applied in the social sciences^2^. However, for the analysis of the cognitive warfare concept, the referential concept model will be used. This is because, the cognitive warfare concept by NATO ACT is a very extensive maximalist concept with a military background that cannot be directly reformatted into the format of the basic concept model without some amount of reconceptualization. However, this would distort the concept analysis. Therefore, the uncomplicated referential concept model is suited much better for a concept analysis in this case since the assignment of elements of the analyzed concept can be allocated to components of the referential concept model is much more straightforward.

Because the quality of concepts is critical to many research endeavors, criteria for the evaluation of concepts have been developed. Sartori's (1970) approach emphasizes the importance of conceptual clarity and precision, advocating for the use of a “ladder of abstraction” to avoid concept stretching in comparative politics. Building on this, Collier and Mahon (1993) refined Sartori's model by offering systematic methods for adjusting conceptual categories, ensuring validity across diverse contexts.

While Goertz's (2002) multi-dimensional model provides a detailed structure for defining complex concepts, it is not suitable for the present conceptual analysis due to its rigid conceptual hierarchy. Adcock and Collier (2001) propose a model that emphasizes the integration of qualitative and quantitative approaches to achieve both content and measurement validity, ensuring that concepts are appropriately operationalized for empirical research. They focus on aligning theoretical definitions with measurable indicators to maintain conceptual rigor.

Transitioning from this, Gerring's model offers a specific set of criteria, such as differentiation, coherence, and utility, that guide the evaluation of concepts, helping to tailor the conceptual framework to the unique needs of my research. Gerring's model is particularly well-suited for the analysis of complex, maximalist concepts due to its comprehensive framework for assessing coherence, differentiation, and utility across diverse contexts. This approach emphasizes the internal structure and definitional clarity of a concept, which is essential for navigating the intricacies and multiple dimensions inherent in maximalist constructs. By focusing on these criteria, Gerring's model facilitates a systematic evaluation that is well-aligned with the complexities typically associated with such expansive conceptual frameworks. In contrast, the model by Adcock and Collier is more focused on ensuring measurement validity and bridging qualitative and quantitative methods, but it may not provide the same depth of analysis needed to unpack and assess the intricate dimensions of a highly complex concept. In his highly cited works on social science methodology Gerring (2001, 2012) has published two frameworks to evaluate concepts: “Criteria of conceptual goodness” as well as “Criteria of conceptualization”. For the application of the evaluation of an already existing concept, the “Criteria of conceptual goodness” (Gerring, 2001) are most suitable.

Gerring (2001) outlined eight criteria for evaluating conceptual goodness: coherence, operationalization, validity, field utility, resonance, contextual range, parsimony, and analytical/empirical utility. Coherence ensures that the concept is internally consistent and free from contradictions, while operationalization emphasizes the ease with which the concept can be translated into measurable indicators for empirical analysis. Validity refers to the extent to which the concept accurately captures the phenomena it is intended to describe. Field utility concerns the concept's practical relevance and usefulness to the field, whereas resonance gauges how intuitively and broadly the concept aligns with existing knowledge or understanding in the discipline. Contextual range assesses the concept's applicability across different settings, ensuring it is versatile without losing its meaning. Parsimony encourages simplicity, aiming for the concept to convey essential ideas without unnecessary complexity. Finally, analytical/empirical utility evaluates the concept's ability to generate meaningful, testable hypotheses and contribute to both theoretical and empirical advancements.

These eight criteria will serve as a comprehensive guideline for evaluating the cognitive warfare exploratory concept as published by NATO ACT (NATO Allied Command Transformation, 2023). By applying each criterion to the concept, a thorough and structured assessment can be conducted, ensuring that the concept's internal structure, practical relevance, and empirical applicability are fully examined. This process aims to provide a coherent evaluation of the cognitive warfare concept's overall quality and utility in the context of scientific research to aid in making the miliary concept of cognitive warfare useable in scientific applications.

### 4.2 Concept evaluation

To methodologically evaluate the cognitive warfare concept, the learnings of the concept review in Section 3 will be assigned to the basic elements of a referential concept. At its core, a concept includes three elements: the term or linguistic label itself, the extension or empirical referent, and the intension, i.e., the defining attributes of a given concept which fill the concept with meaning (Sartori, 1984; Gerring, 2001; Wonka, 2007).

The term of the concept is cognitive warfare, thereby clarifying that the concept describes a type of warfare happening in the cognitive domain/dimension. The defining attributes, that fill the concept with meaning (intension or connotation) are divided in two categories. The first set of attributes that constitute cognitive warfare are its operations and tactics, which are traditional vectors and enablers, existing technology vectors and enablers as well as emerging technology vectors and enablers. The second set of attributes are the intended effects of cognitive warfare, which are impeding decision-making and the disruption of the Observe, Orient, Decide, Act (OODA) loop, the division and polarization of societies, weaponizing identity, weaponizing narratives and impacting the will to fight.

The concept's extension, or its empirical referents, poses a greater challenge to apprehend compared to its defining attributes. This complexity arises from the evolving nature of cognitive warfare and the nascent stage of many technologies outlined in the exploratory concept. As a result, pinpointing precise instances of cognitive warfare proves problematic; cases of cognitive warfare in the contemporary scientific literature often follow a radically different conceptualization of cognitive warfare (see for example 7). Consequently, a theoretical scenario of cognitive warfare is inferred from the exploratory concept. Here it can be deduced that the extension of the concept could be a series of synchronized cognitive attacks, defined as “offensive actions employed to achieve effects on perceptions, beliefs, interests, aims, decisions and behaviors by deliberately targeting the human mind” (NATO Allied Command Transformation, 2023) in the information environment, using EDTs, in individuals, groups or societies, which are particularly vulnerable to cognitive attacks.

After assigning the elements of the cognitive warfare exploratory concept to the basic elements of a referential concept, the next step is its evaluation using Gerring's (2001) eight criteria for conceptual goodness. The first criterion is coherence, which inquires how internally coherent and externally differentiated a concept's attributes are regarding neighboring concepts. For cognitive warfare, the internal coherence can be considered high because the different attributes build upon each other to characterize the clearly defined mechanisms. The cognitive warfare exploratory concept meticulously outlines several measures and effects that are considered to be cognitive warfare, in sum forming a coherent concept. As far as the external differentiation goes, the concept differs from neighboring concepts in several key issues, namely the focus on cognitive effects and actions in the information environment using EDTs, and the sector specific focus on the military and the protection of the MIoP, the concept is therefore sufficiently coherent. However, it could be argued that many attributes associated with cognitive warfare might equally apply to adjacent concepts such as hybrid threats or hybrid warfare, posing an analytical challenge. This issue could be resolved in two ways. Firstly, by acknowledging that cognitive warfare may be too akin to the established concepts of hybrid threats and hybrid warfare, implying it offers limited analytical utility or merely serves as a subordinate tactic within these broader concepts. Alternatively, the cognitive warfare concept could undergo reconceptualization, emphasizing those features that distinctly set it apart from related concepts. This would likely entail a sharper focus on Emerging Disruptive Technologies (EDTs) and cognitive processes and effects that extend beyond well-known strategies such as disinformation, propaganda, and information operations.

The second criterion, operationalization, probes the concept's ability to differentiate its own referents from other empirical referents distinctly. In this regard, the cognitive warfare concept faces a number of challenges. First, as the concept is concerned with emerging technologies, which is on reason why it might currently lack concrete instances in the field. Second, the concept is inherently future oriented, which means that is not aimed to measure present day instances. Lastly, due to the covert nature of many tactics associated with cognitive warfare, detecting its occurrence may be challenging, even if they were to happen. Classifying a conflict or power competition as cognitive warfare involves assessing whether it meets specific thresholds. This approach is consistent with the measurement of many complex concepts in the social sciences, where determining the presence of a phenomenon often requires evaluating multiple dimensions. For measuring cognitive warfare, it could be argued that some present conflicts, like recent Russian activities in the Baltic (Oksanen et al., 2024), Chinas behavior toward Taiwan and its activities in the Taiwan Strait (Hung and Hung, 2022), Chinas activities in the South China Sea dispute (Hong, 2013) as well as the Russian warfare in its war on Ukraine and the Ukrainian defensive effort (Muradov, 2022; Dov Bachmann et al., 2023) could be classified as cognitive warfare, provided all essential attributes of the concept are present. From a quantitative perspective, the challenge lies in determining whether the intensity and scale of these observed activities sufficiently meet the thresholds required to identify each necessary attribute of cognitive warfare in these specific cases.

The third criterion, validity, addresses whether the concept accurately measures what it is intended to represent. This evaluation is challenging given the difficulties of classifying current conflicts or power competitions as cognitive warfare with a maximalist concept like the NATO ACT concept. It is pertinent to note once more, that the concept is future-oriented, and present-day instances would either be classified under different conceptual frameworks or the threshold of classifying a given case as cognitive warfare would need to be adjusted. Therefore, it can be concluded that identifying and measuring concrete instances of cognitive warfare using the NATO ACT concept in the field is challenging. This may change with further advancements in EDTs, which play in important role in cognitive warfare. However, a slight reconceptualization can change this outlook ^2^.

The fourth criterion, field utility, assesses the practical usefulness of the concept in comparison to similar ones. Currently, in the realm of cognitive warfare, concepts like hybrid threats, hybrid warfare or information warfare hold greater analytical utility. However, as technological capacities continue to advance, cognitive warfare has the potential to offer a significant contribution in comprehending forthcoming threats more effectively. The concept does indeed describe a novel, emerging threat, which has previously not been described by existing concepts.

The fifth criterion, resonance, examines whether the concept holds relevance in both general and specialized contexts. Within NATO and military circles, the concept of cognitive warfare finds resonance, because of its function in official NATO doctrine. However, in broader non-military contexts, the explicit military focus and the use of the term “warfare” in cognitive warfare may complicate communication efforts. Established concepts like hybrid threats are more commonly used in these scenarios, particularly because terms incorporating “warfare” tend to be less palatable to the general public. However, the concept might be an effective tool to analytically focus analyses on cognitive effects in a diverse set of adversarial measures. While the cognitive warfare concept by NATO ACT is designed to be a concept that shall help to develop tactics to defend NATO against potential cognitive warfare by adversaries, a potential drawback of the term cognitive warfare could also be, that the term could be misperceived in the general public or even misused in adversarial disinformation.

The sixth criterion, contextual range, assesses the concept's applicability across different languages. “cognitive warfare” is a term that distinctly conveys its meaning and can be meaningfully translated. However, in other strategic cultures other concepts exist, such as Russian reflexive control (Jaitner and Kantola, 2016) and Chinese indirect approaches (Aukia and Kubica, 2023) or the three warfare strategy (Lee, 2014), which partially overlap with cognitive warfare, making the concept less meaningful in these cultural contexts.

The seventh criterion, parsimony, evaluates the conciseness of the term and its list of attributes. The term “cognitive warfare” itself is succinct and precise. However, its attributes in the NATO ACT concept are extensive and complex, as they are feature an long list of measures and effects, which form a complex maximalist concept. The concept includes many measures and effects because it is a military concept, which must be linked to specific defense capabilities. For a scientific application the concept could benefit from a reduced list of attributes, which focuses on the most important measures and effects, while removing some attributes which are analytically less relevant.

The eighth criterion, analytic/empirical utility, pertains to how useful the concept is in analytic contexts and research designs. In contexts focused on emerging threats, “cognitive warfare” holds significant analytical potential. It could serve as a tool to grasp and conceptualize several possible cognitive effects of many adversarial measures in conflicts below and above the threshold of war. Nevertheless, the concept's applicability in contemporary empirical research remains constrained. This limitation arises from the broad array of attributes, the emphasis on emerging disruptive technologies (EDTs), and the necessity for the concurrent application of various measures. Collectively, these factors establish an exceedingly high benchmark for classifying a scenario as cognitive warfare, rendering the concept impractical for empirical analysis.

## 5 Discussion

The evaluation of the cognitive warfare concept based on John Gerring's eight criteria for conceptual goodness reveals several insights. First the lack of real-world instances of cognitive warfare as defined by NATO ACT complicates the validation of the concept and its current practical value, yet its forward-looking nature is in keeping with its intended purpose. Although hybrid threats and hybrid warfare currently offer greater practical utility, cognitive warfare is expected to become increasingly pertinent as technological advancements continue, thereby enhancing its future relevance in the field.

As mentioned above, the term “cognitive warfare” is clear and translatable across languages. However, the term may be difficult in different strategic cultures. Also, the introduction of a concept with the term “warfare” in the title may be problematic in some political and societal arenas. While concise, the list of attributes may require further elaboration. Lastly, in contexts focused on emerging threats, the concept holds significant analytical potential, though its current empirical applicability may be limited.

The concept demonstrates high internal coherence, as its attributes synergistically characterize defined mechanisms. However, the external differentiation from neighboring concepts can potentially be problematic. The concepts unique features lie in analyzing potential cognitive effects through the use of EDTs, and its specific focus on the military sector and the protection of the MIoP. In the existing conceptual landscape, it is debatable whether cognitive warfare needs to be defined as a standalone concept. Instead, it could be more effectively conceptualized as a subordinate concept—or tactic—within the frameworks of hybrid threats or hybrid warfare. This approach could address many of the conceptual challenges associated with cognitive warfare, particularly those related to extensive lists of attributes and conceptual stretching.

Furthermore, the concept faces challenges in operationalization, as concrete instances in the field are currently lacking. This is partly due to the extensive list of defining attributes and an inherent focus on emerging technologies. Furthermore, the covert nature of many associated tactics makes detection difficult. In the literature, more streamlined conceptualizations of cognitive warfare have been introduced. This presents a trade-off: while leaner concepts are generally more suitable for social scientific research and political communication, they may offer limited utility in military contexts. This is because such conceptualizations describe fewer capabilities and might omit critical attack vectors. A practical approach would be to develop a smaller, more focused concept that is interoperable with the broader, more comprehensive frameworks. This conceptualization of cognitive warfare would therefore need to be interoperable with the expansive military concept of cognitive warfare by NATO ACT, while also aligning with contemporary literature on hybrid threats or hybrid warfare.

Above we have introduced a working definition of cognitive warfare, which builds upon existing literature and the concept by NATO ACT: *Cognitive warfare is a tactic, which combines traditional and emerging technologies as well as measures above and below the threshold of war to achieve cognitive effects in an adversary's population, as well as in their political and military leaders*. In order to demonstrate how the cognitive warfare concept by NATO ACT could be reconceptualized be gain more analytical value in a scientific application by streamlining it, we visualize our working definition of cognitive warfare, using the basic concept model by Goertz (2002).

**Figure 1:**
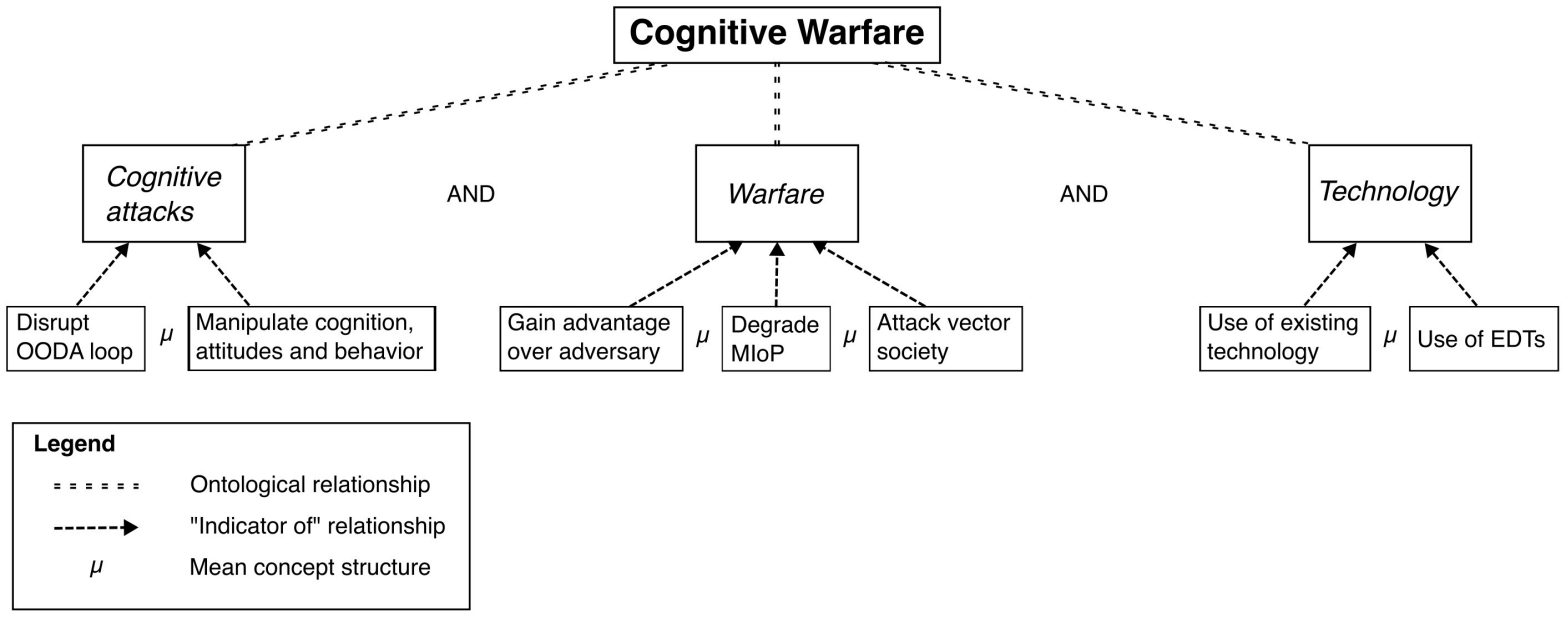
Visualization of the cognitive warfare working definition.

Level 1, or the basic level in Figure 1, represents the core concept of cognitive warfare. As previously discussed, our working definition of cognitive warfare includes three essential elements: achieving cognitive effects, elements of warfare, and the utilization of technology. This basic level outlines the broad, foundational idea of cognitive warfare, serving as an entry point into the concept's more detailed structure.

At Level 2, or the secondary level, we see the breakdown of the concept into its core attributes. These attributes are ontologically interconnected, meaning they work together to give the concept its specific meaning and function. For our working definition, the three attributes—cognitive effects, warfare elements, and technology—are necessary conditions; all must be present in any given case to classify it as an instance of cognitive warfare. The attributes at this level offer a more precise framework for understanding the internal structure of the concept.

Level 3, the data indicator level, focuses on the empirical indicators that suggest the presence of the attributes outlined in Level 2. Here, the concept adopts what Goertz (2002) refers to as a “mean concept structure,” meaning that not every indicator needs to be present for an attribute to be identified. Instead, a subset of indicators may suffice, as long as the cumulative evidence surpasses a predetermined threshold. In other words, while the concept's attributes are necessary, the empirical evidence for each attribute need not be exhaustive—only sufficiently robust to suggest the presence of the attribute. This tiered structure allows for a nuanced application of the cognitive warfare concept, where the intensity and combination of indicators at Level 3 guide the assessment of whether a given case qualifies under the overarching framework at Level 1.

Our working definition of cognitive warfare exemplifies how a concept can be reconceptualized to enhance its value for scientific applications while maintaining its core integrity and interoperability with NATO ACT's framework. By focusing on three essential elements—achieving cognitive effects, elements of warfare, and the use of technology—we streamline the NATO ACT definition, making it more operationalizable and empirically testable without sacrificing the essence of the concept. This refined structure preserves the emphasis on the military sector and cognitive effects while addressing the challenges of its broader, more abstract attributes. Importantly, our reconceptualization maintains compatibility with NATO ACT's approach, ensuring that the concept remains interoperable in both military and strategic contexts, while also being better suited for academic analysis. This balance between clarity, coherence, and operational utility strengthens the concept's relevance for both scientific inquiry and real-world applications.

## 6 Conclusion

In conclusion, the conceptualization of cognitive warfare presents both challenges and opportunities for military and analytical applications. The analysis reveals that the cognitive warfare concept by NATO ACT is an inherently future oriented concept, which can be classified as a maximalist approach to the conceptualization of cognitive warfare. There is considerable conceptual overlap between cognitive warfare and neighboring concepts such as Foreign Information Manipulation and Interference (FIMI), hybrid threats, and hybrid warfare, given that cognitive warfare shares numerous attributes with these established frameworks. This overlap suggests that cognitive warfare could be effectively integrated as a tactical element within broader hybrid threat strategies, thereby enhancing the analytical depth and understanding of hybrid effects by situating cognitive operations within an established, multifaceted context. The present maximalist conceptualization must undergo a reconceptualization and streamlining to improve its utility in research applications. Any concept resulting from a reconceptualization must be interoperable, aligning with both the comprehensive military perspective of cognitive warfare and the academic insights provided by hybrid threats literature. Above we presented an option how a maximalist cognitive warfare concept could be reconceptualized to make it empirically measurable and improve its analytic utility.

Cognitive warfare has a notable focus on the use of Emerging Disruptive Technologies (EDTs) and the resulting cognitive effects. This innovation highlights the concept's relevance in modern warfare where technological advancements play a pivotal role. However, the empirical analysis of cognitive warfare is currently hampered by a lack of instances in the field and the challenges of measurement. Despite these difficulties, the concept holds considerable potential for analyses focused on foresight and capacity building, where its forward-looking nature can provide significant insights. Furthermore, its distinct focus on cognitive effects could potentially enhance the explanatory power of hybrid threats frameworks when used as a subordinate concept of hybrid threats or hybrid warfare.

Within the NATO doctrine, the cognitive warfare concept will fulfill its function as a lower-level military concept. Given its focus on rapidly evolving EDTs, it is likely to require frequent updates and modifications to remain effective and relevant. This need for continual adaptation speaks to the dynamic nature of cognitive warfare and the fast-paced technological environment in which it operates.

The NATO ACT's conceptualization of cognitive warfare represents a maximalist definition, incorporating a broader range of attributes compared to more minimalist interpretations found in the literature. This broader approach, as developed by NATO ACT, allows for a more comprehensive application in strategic military planning and operations. However, it also necessitates a clear understanding and delineation of cognitive warfare to prevent conceptual stretching and ensure its effective integration into military and analytical frameworks.

By considering these aspects, it becomes evident that while cognitive warfare is a potent and evolving concept, its successful implementation and utility depend on careful consideration of its scope and the context in which it is applied. As we move forward, it will be crucial to continue refining the concept to ensure it remains a valuable tool in the arsenal of modern military strategy and analysis.

## Footnotes

^1^Annex C of the NATO ACT cognitive warfare exploratory concept is an attempt at an actor and value neutral taxonomy of cognitive warfare. However, as the authors themselves state, a significant overlap between different measures exists. Since the taxonomy offers little analytical potential, it is not subject to analysis in the present work. Furthermore, a highly differentiated taxonomy of an already extensive concept like cognitive warfare poses a high risk of an over specification of the concept.

^2^See Section 5.
